# Comparative genomic analysis of four representative plant growth-promoting rhizobacteria in *Pseudomonas*

**DOI:** 10.1186/1471-2164-14-271

**Published:** 2013-04-22

**Authors:** Xuemei Shen, Hongbo Hu, Huasong Peng, Wei Wang, Xuehong Zhang

**Affiliations:** 1 State Key Laboratory of Microbial Metabolism, School of Life Sciences and Biotechnology, Shanghai Jiao Tong University, 800 Dongchuan Road, Shanghai, 200240, People’s Republic of China

**Keywords:** Plant growth-promoting rhizobacteria, Pseudomonad, Comparative genomics, Environmental adaptability, Rhizosphere colonization, Biocontrol activity

## Abstract

**Background:**

Some *Pseudomonas* strains function as predominant plant growth-promoting rhizobacteria (PGPR). Within this group, *Pseudomonas chlororaphis* and *Pseudomonas fluorescens* are non-pathogenic biocontrol agents, and some *Pseudomonas aeruginosa* and *Pseudomonas stutzeri* strains are PGPR. *P*. *chlororaphis* GP72 is a plant growth-promoting rhizobacterium with a fully sequenced genome. We conducted a genomic analysis comparing GP72 with three other pseudomonad PGPR: *P*. *fluorescens* Pf-5, *P*. *aeruginosa* M18, and the nitrogen-fixing strain *P*. *stutzeri* A1501. Our aim was to identify the similarities and differences among these strains using a comparative genomic approach to clarify the mechanisms of plant growth-promoting activity.

**Results:**

The genome sizes of GP72, Pf-5, M18, and A1501 ranged from 4.6 to 7.1 M, and the number of protein-coding genes varied among the four species. Clusters of Orthologous Groups (COGs) analysis assigned functions to predicted proteins. The COGs distributions were similar among the four species. However, the percentage of genes encoding transposases and their inactivated derivatives (COG L) was 1.33% of the total genes with COGs classifications in A1501, 0.21% in GP72, 0.02% in Pf-5, and 0.11% in M18. A phylogenetic analysis indicated that GP72 and Pf-5 were the most closely related strains, consistent with the genome alignment results. Comparisons of predicted coding sequences (CDSs) between GP72 and Pf-5 revealed 3544 conserved genes. There were fewer conserved genes when GP72 CDSs were compared with those of A1501 and M18. Comparisons among the four *Pseudomonas* species revealed 603 conserved genes in GP72, illustrating common plant growth-promoting traits shared among these PGPR. Conserved genes were related to catabolism, transport of plant-derived compounds, stress resistance, and rhizosphere colonization. Some strain-specific CDSs were related to different kinds of biocontrol activities or plant growth promotion. The GP72 genome contained the *cus* operon (related to heavy metal resistance) and a gene cluster involved in type IV pilus biosynthesis, which confers adhesion ability.

**Conclusions:**

Comparative genomic analysis of four representative PGPR revealed some conserved regions, indicating common characteristics (metabolism of plant-derived compounds, heavy metal resistance, and rhizosphere colonization) among these pseudomonad PGPR. Genomic regions specific to each strain provide clues to its lifestyle, ecological adaptation, and physiological role in the rhizosphere.

## Background

*Pseudomonas* (sensu stricto) is a diverse genus that occupies many different niches and exhibits versatile metabolic capacity [1]. A number of pseudomonad strains function as plant growth-promoting rhizobacteria (PGPR). Such strains can protect plants from various soilborne pathogens and/or stimulate plant growth [2]. For example, *Pseudomonas chlororaphis* and *Pseudomonas fluorescens* are non-pathogenic biocontrol agents, while several strains of *Pseudomonas aeruginosa* and *Pseudomonas stutzeri* show strong plant growth-promoting activities. Some characteristic features associated with plant growth promotion have been studied at the molecular level. For example, effective PGPR show sufficient colonization of the rhizosphere [3,4]. Moreover, PGPR have certain biocontrol activities; for example, they can produce antibiotics that prevent infection by plant pathogens [2]. Such antibiotics include phenazine derivatives [5-7], pyoluteorin (Plt) [8,9], pyrrolnitrin (Prn) [10], hydrogen cyanide (HCN) [11], and so on. Some rhizobacteria directly promote plant growth in the absence of pathogens [12]. However, a comprehensive analysis of the characteristics of PGPR among different *Pseudomonas* species using a comparative genomics approach has not been reported yet.

Comparative genomics has emerged as a powerful tool to identify functionally important genomic elements [13-15]. As more and more genomic information becomes available, the development of genomic technologies can provide further insights into essential life processes [16]. As of March 2012, the complete genomic sequences of strains representing the following *Pseudomonas* species were available: the rhizobacteria *P*. *fluorescens*[14,17], *P*. *stutzeri*[18], and *Pseudomonas putida*[15,19,20], and the pathogens *P*. *aeruginosa*[21,22], *Pseudomonas syringae*[23], and *Pseudomonas entomophila*[24]. Genomic information for *P*. *chlororaphis*, which plays an important role in suppressing pathogens and stimulating plant growth [3,25-27], was first reported by our group [28].

*P*. *chlororaphis* GP72 (hereafter, GP72) was isolated from the rhizosphere of green pepper in China. This strain shows broad antagonistic activities [29]. It completely suppresses the phytopathogens *Coletotrichum lagenarium*, *Pythium ultimum*, *Sclerotinia sclerotiorum*, *Fusarium oxysporum* f. sp. *cucumerinum*, *Carposina sasakii*, and *Rhizoctonia solani*[7]. In vitro screening and in vivo genetic engineering experiments showed that GP72 can produce two phenazine derivatives, phenazine-1-carboxylic acid (PCA) and 2-hydroxyphenazine (2-OH-PHZ), which have strong fungicidal activities [30]. It also produces other secondary metabolites including HCN, indole-3-acetic acid (IAA), and siderophores, contributing to rhizosphere adaptability [7]. *P*. *fluorescens* Pf-5 (hereafter, Pf-5) was isolated from the rhizosphere of cotton and is able to suppress damping-off caused by *P*. *ultimum*[8]. As a well-recognized biocontrol agent, Pf-5 produces a spectrum of antibiotics including Prn, Plt, and 2,4-diacetylphloroglucinol (DAPG) [8,31], and two siderophores, pyoverdine (Pvd) and pyochelin (Pch). *P*. *aeruginosa* M18 (hereafter, M18) was isolated from the rhizosphere of sweet melon. It produces both PCA and Plt, which show strong antifungal activities [22,32], and it shows biocontrol activity in the rhizosphere niche [33]. Most *P*. *aeruginosa* strains are opportunistic pathogens, including the first-sequenced strain, *P*. *aeruginosa* PAO1 [21,34,35]. *P*. *stutzeri* A1501 (hereafter, A1501), which belongs to the nonfluorescent *Pseudomonas* group, was isolated from the rhizosphere of rice based on its nitrogen-fixation capacity [36,37]. A1501 has been commercialized for use as a crop inoculant in China. Genetic information for A1501 has provided insights into the mechanisms of its nitrogen-fixation ability and environmental adaptability, such as its ability to mineralize aromatic compounds [18,38].

To identify the shared characteristics of pseudomonad PGPR, we compared genomic information for GP72 with those of three other representative pseudomonad PGPR: the biological control agent Pf-5, the rhizobacterium M18, and the nitrogen-fixing strain A1501. There were 602 genes conserved among the four species. Comparison among these PGPR also revealed previously unknown common traits related to plant growth promotion. This comparative genomics analysis of different PGPR provides information about the genetic basis of diversity and adaptation. The results of this study also provide foundation knowledge to improve and exploit the plant growth-promoting activities of PGPR in agricultural applications via molecular techniques.

## Results and discussion

### General genome features and comparative genomics

**Figure 1:**
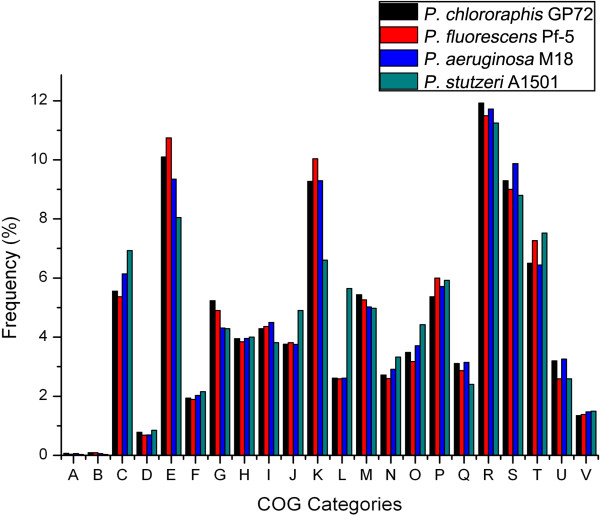
**Comparison of COG categories among four pseudomonad PGPR.** Functional classifications provided by the COG database [40] were used for functional comparisons among the genomes of *P*. *chlororaphis* GP72, *P*. *fluorescens* Pf-5, *P*. *aeruginosa* M18, and *P*. *stutzeri* A1501. The ordinate axis indicates the percentage of genes in each COG functional category relative to the genes of all COG categories. Comparison was based on 22 COGs categories: RNA processing and modification (**A**), chromatin structure and dynamics (**B**), energy production and conversion (**C**), cell cycle control, cell division, chromosome partitioning (**D**), amino acid transport and metabolism (**E**), nucleotide transport and metabolism (**F**), carbohydrate transport and metabolism (**G**), coenzyme transport and metabolism (**H**), lipid transport and metabolism (**I**), translation, ribosomal structure and biogenesis (**J**), transcription (**K**), replication, recombination and repair (**L**), cell wall, membrane, envelope biogenesis (**M**), cell motility (**N**), posttranslational modification, protein turnover, chaperones (**O**), inorganic transport and metabolism (**P**), secondary metabolites biosynthesis, transport and catabolism (**Q**), general function prediction only (**R**), function unknown (**S**), signal transduction mechanisms (**T**), intracellular trafficking, secretion and vesicular transport (**U**), defense mechanisms (**V**).

The general features of the four PGPR genomes are summarized in Table 1. The assembled genome of GP72 had approximately 270-fold sequence coverage [28], with putative functions assigned to 83% of the genes. It is reasonable to assume that the vast majority of genes are important for cell metabolism. GP72, Pf-5, M18, and A1501 showed a wide range of genome sizes, ranging from 4.6 to 7.1 M, resulting in different numbers of protein-coding genes (Table 1). The genome sequences and additional information related to each predicted gene, such as gene product annotation, KEGG orthology, gene ontology, and predicted subcellular location are available on the IMG database (https://img.jgi.doe.gov/cgi-bin/er/main.cgi) [39]. Predicted proteins were functionally categorized using the COGs database [40], and COGs categories were compared among the genomes of GP72, Pf-5, M18, and A1501 (Figure 1). The COGs showed similar distributions among the four strains, except for the COGs K and L, which were quite different in A1501 compared with the other strains. The percentage of genes with COG K annotations, representing transcription clusters, was lower in A1501 than in the other three strains, mainly because it contained a smaller proportion of genes encoding transcriptional regulators. COG L represents proteins with functions in replication, recombination, and repair. There were 50 genes encoding transposases and their inactivated derivatives in A1501 (approximately 1.33% of the total genes with COG annotations), compared with 0.21% in GP72, 0.02% in Pf-5, and 0.11% in M18. The large number of transposases in A1501 indicated that this strain would be more suitable for transposition, providing clues to the genetic diversity within this species and its adaptability to changes in growth conditions.

**Table 1:** General genome features of the four studied pseudomonad PGPR

	**GP72**	**Pf-5**	**M18**	**A1501**
Size (base pairs)	6,663,241	7,074,893	6,327,754	4,567,418
G+C content (%)	63.13%	63.30%	66.50%	63.88%
Protein-coding genes	6091	6142	5690	4135
No. of protein-coding genes with function prediction	5062 (83.11%)	4492 (73.14%)	4115 (72.32%)	3227 (78.04%)
No. of protein-coding genes without function prediction	16.89%	26.86%	27.68%	21.96%
No. of protein-coding genes connected to KEGG Orthology	53.77%	51.84%	53.95%	57.61%
No. of protein-coding genes with COGs	82.66%	79.27%	83.88%	80.58%
No. of protein-coding genes coding signal peptides	24.48%	24.28%	25.17%	22.03%
No. of protein-coding genes coding transmembrane proteins	23.71%	23.58%	23.71%	24.76%
RNA genes	85	115	79	102
rRNA genes (5S rRNA, 16S rRNA, 23S rRNA)	4 (2, 1, 1)	16 (6, 5, 5)	13 (5, 4, 4)	13 (4,4,5)
tRNA genes	61	71	61	61
Other RNA genes	20	28	5	28
Conserved CDS	602	558	572	545
Strain-specific CDS	994	1116	1351	1195

Gene annotations and comparisons were obtained from IMG database [39]. Numbers of conserved and specific genes in each strain determined by comparison to other PGPR genomes. Genes with homology (H) values less than 0.42 and more than 0.81 were arbitrarily defined as specific and conserved, respectively.

**Figure 2:**
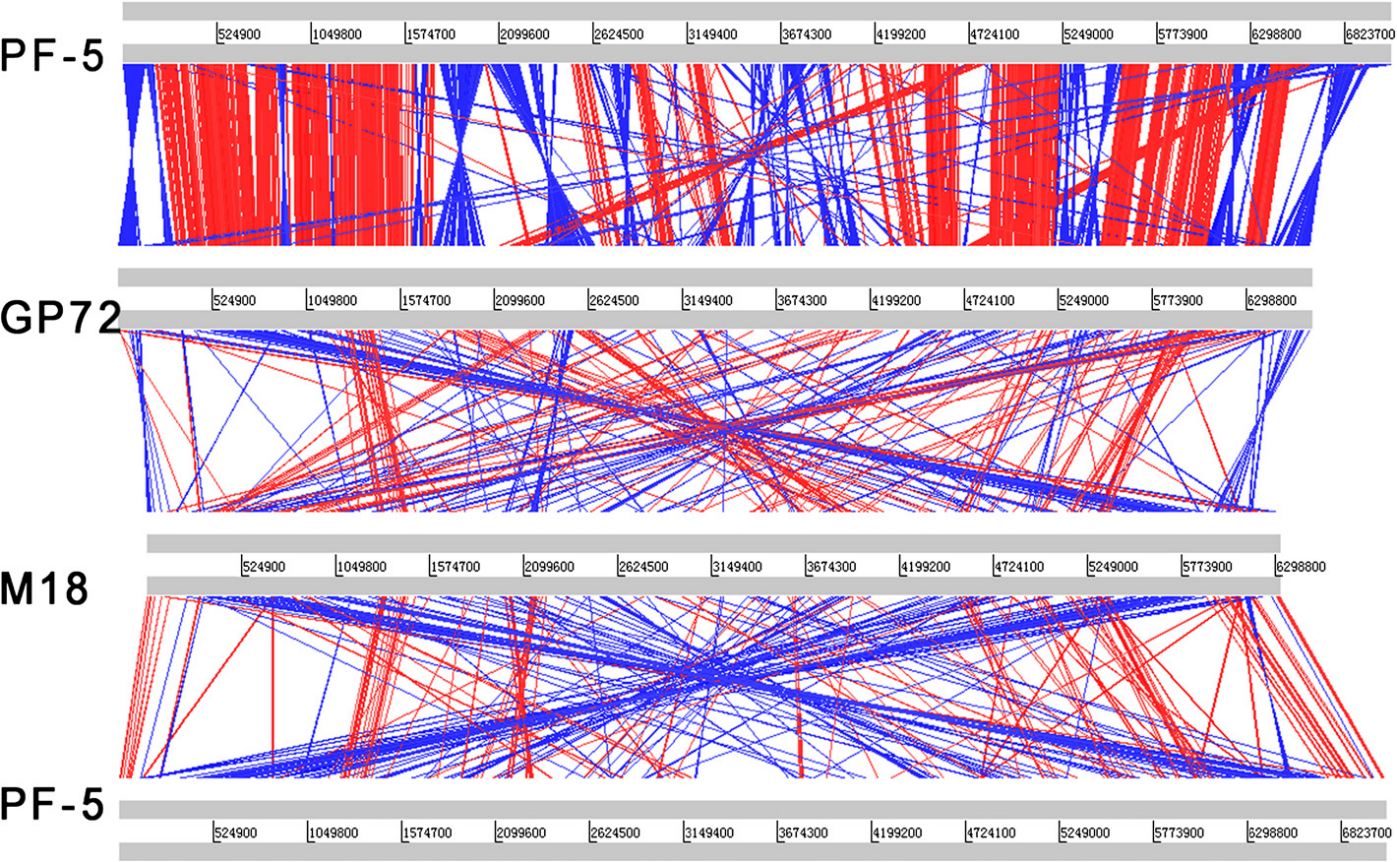
**Comparison of chromosome structures among genome sequences of pseudomonad PGPR.** Pair-wise alignments between genome sequences of *P*. *fluorescens* Pf-5, *P*. *chlororaphis* GP72, and *P*. *aeruginosa* M18 were performed using WebACT [41]. Red bars indicate collinear regions of similarity; blue bars represent regions of similarity that have been inverted in one of the two genomes. Only matches larger than 1 kb are shown.

**Figure 3:**
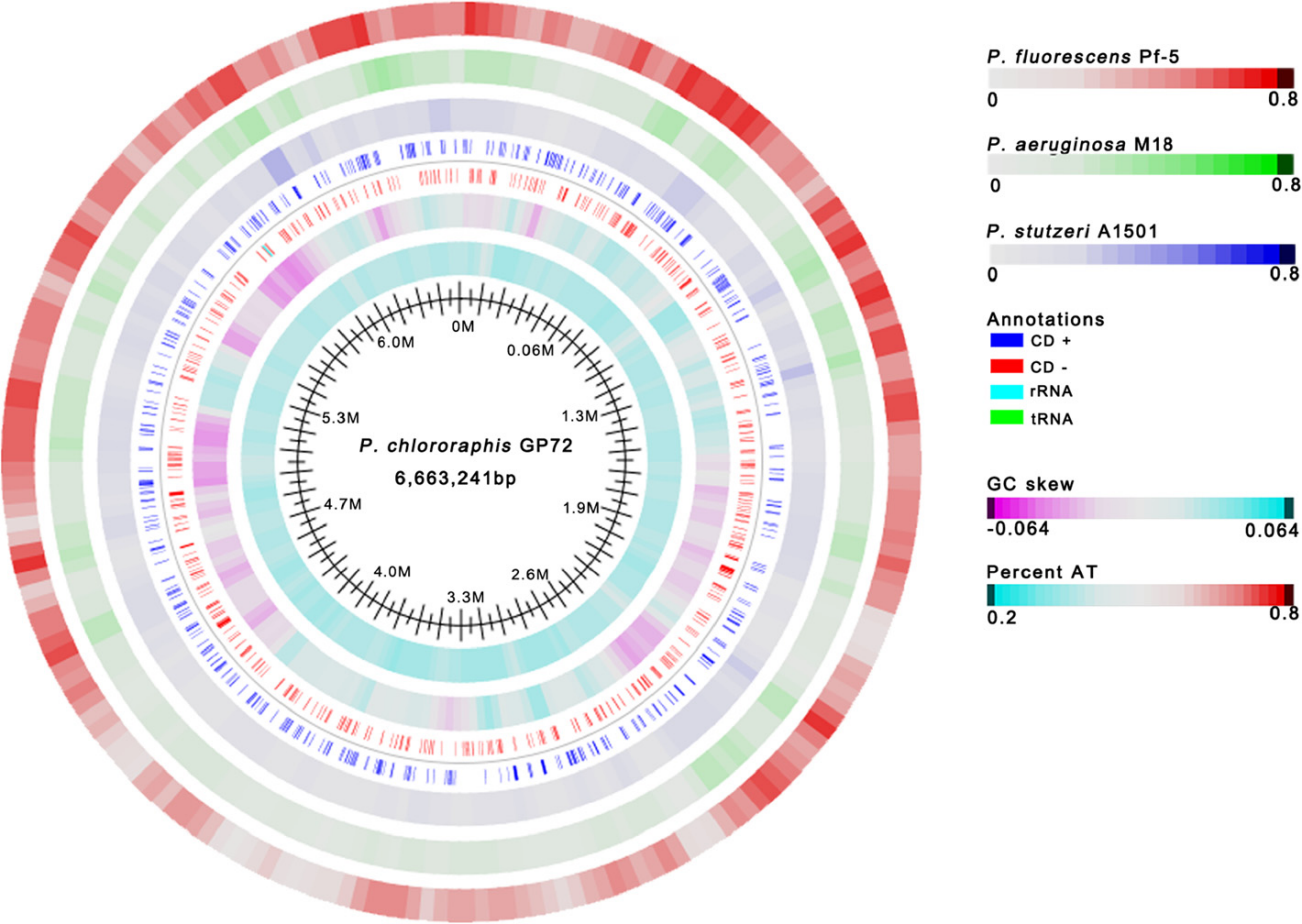
**BLAST atlas diagram showing homology among pseudomonad PGPR.** Comparisons between *P*. *chlororaphis* GP72 and three other pseudomonad PGPR. Colors indicate strains, as follows (starting from the outermost line): red, *P*. *fluorescens* Pf-5 (line 1); green, *P*. *aeruginosa* M18 (line 2); blue, *P*. *stutzeri* A1501 (line 3). Lack of color indicates that genes at that position in GP72 were absent from genome of strain in that line. Predicted CDSs of reference genome (GP72) on plus and minus strand are shown as blue and red blocks; rRNA genes are shown in green, tRNA genes are shown in turquoise. GC skew (line 6) and percent AT (line 7) are also shown.

Global alignments provide a powerful tool to identify conserved and specific regions in the genome, which can reveal similar biological behaviors or adaptations to specific niches. We conducted BLASTN analysis using an online version of the Artemis Comparison Tool (WebACT) [41], comparing GP72, Pf-5, and M18 (Figure 2). We excluded A1501 from this analysis, since the alignment analysis showed very low synteny (data not shown). According to alignments at current assemblies, the extent of conservation of regions among the different species of *Pseudomonas* was difficult to visualize by ACT, mainly because of multiple chromosomal rearrangements. Nevertheless, the genomes of Pf-5 and GP72 showed many regions with conserved sequences and conserved gene order, except for 10 major inversions. BLAST atlas, which provides a quick overview of genomic regions of gene conservation across many genomes [42], was used to compare the reference genome of GP72 to the other three query genomes (Figure 3).

**Figure 4:**
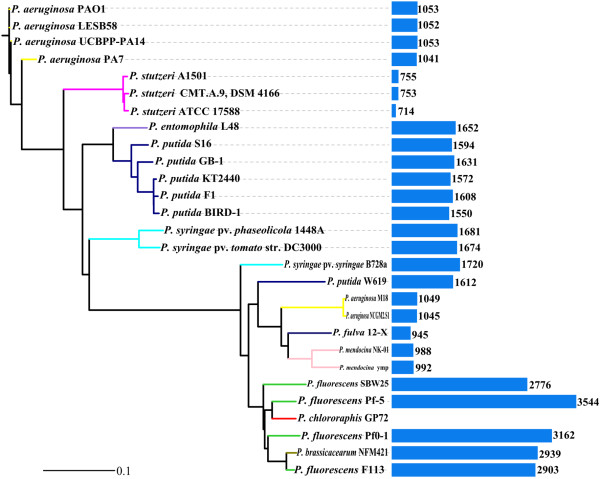
**Phylogenetic relationships among completely sequenced *Pseudomonas *species.** Phylogenetic tree for members of the genus *Pseudomonas* was constructed based on aligned concatenated sequences of *gyrB* and *rpoD* using the neighbor-joining method with 1000 bootstrap replicates. Analysis was carried out using Phylip 3.67 software and the tree was plotted using iTOL software. Colors on the phylogenetic tree indicate membership in *Pseudomonas* phylogenetic groups according to NCBI taxonomy. Completely sequenced species in the genus *Pseudomonas* include *P*. *aeruginosa* (yellow), *P*. *brassicacearum* (olive), *P*. *entomophila* (purple), *P*. *fluorescens* (green), *P*. *fulva* (blue), *P*. *mendocina* (pink), *P*. *putida* (navy), *P*. *stutzeri* (magenta), and *P*. *syringae* (cyan). In this research, the tree branch of *P*. *chlororaphis*, whose draft genome sequence was reported recently, is shown in red. Bar chart associated with nodes indicates numbers of genes conserved between GP72 and the corresponding organism. Conserved genes were determined using mGenomeSubtractor.

We established a phylogenetic tree of completely sequenced pseudomonads based on two housekeeping genes (*gyrB* and *rpoD*) (Figure 4). The tree showed that GP72 was most closely related to *P*. *fluorescens*, and was more closely related to *P*. *aeruginosa* M18 than to other opportunistic pathogenic strains of *P*. *aeruginosa*. This was probably because the rhizosphere-originated M18 strain has evolved strain-specific genomic features, which benefit its environmental adaptability and competitiveness under certain conditions in the rhizosphere niche.

**Figure 5:**
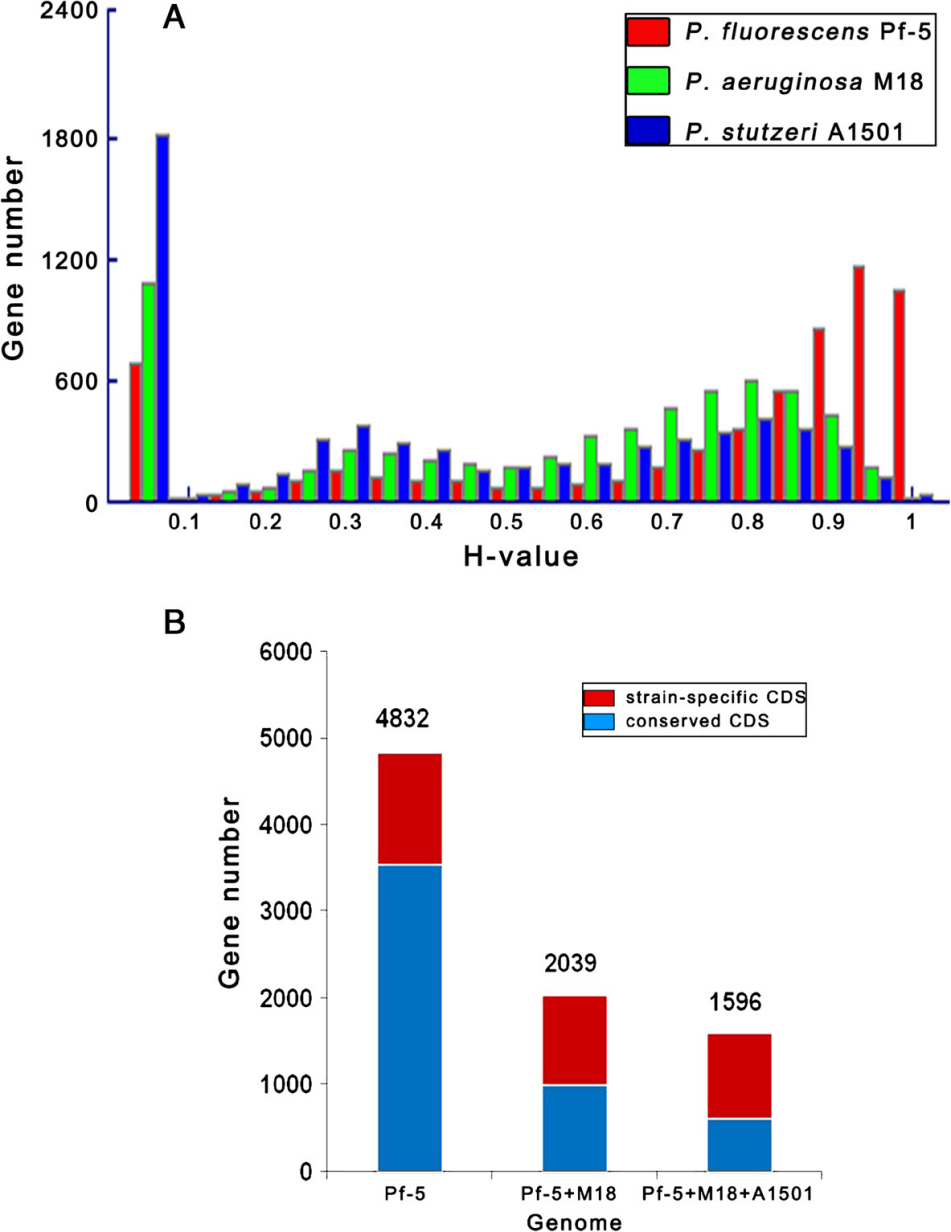
**Homology analysis between *P. chlororaphis *GP72 genome and three subject genomes.** The mGenomeSubtractor arbitrarily defines CDSs with homology (H) values less than 0.42 as strain-specific, and those with H values greater than 0.81 as conserved [43]. (**A**) Histogram of BLASTP-based homology value distribution of 6091 predicted CDSs from *P*. *chlororaphis* GP72 compared individually with those of three other genomes: *P*. *fluorescens* Pf-5, *P*. *aeruginosa* M18, and *P*. *stutzeri* A1501. (**B**) Numbers of conserved and specific genes in GP72 compared with three other PGPR strains. Total numbers of conserved and specific genes are shown above columns.

An *in silico* subtractive hybridization analysis using the mGenomeSubtractor web server identified specific and conserved proteins. In this analysis, proteins with homology (H) values of less than 0.42 or more than 0.81 are defined arbitrarily as specific or conserved, respectively [43]. The BLASTP-based homology value distribution of 6091 predicted CDSs from *P*. *chlororaphis* GP72 was individually compared with those of the other three subject genomes (Figure 5A) to determine the degree of protein conservation between GP72 and each of the other genomes. Among the genes encoded in the GP72 genome, 3,544 had counterparts in the genome sequence of Pf-5. There were 999 genes conserved among the genomes of GP72, Pf-5, and M18. Comparison among GP72 and all of the other three strains (Pf-5, M18, and A1501) revealed 602 homologous genes and 994 CDSs that were strain-specific to GP72 (Figure 5B). The number of homologs in each genome is shown in Figure 4. In addition, GP72 contained 463 CDSs that were identified as strain-specific (E-value <10^-5^) when its genome was compared with those of 27 other completely sequenced *Pseudomonas* strains. Further analyses of these strain-specific CDS may provide clues to the phenotype and the specific environmental adaptations of each strain.

### Environmental adaptability

#### Catabolism

PGPR can use a wide range of nutrients to colonize the rhizosphere successfully. The central metabolic pathways in GP72, such as the Entner–Doudoroff pathway, the pentose phosphate pathway, and the tricarboxylic acid cycle, are consistent with those reported for other *Pseudomonas* species [44]. Like other *Pseudomonas* strains, GP72 lacks 6-phosphofructokinase; therefore, it may not have a functional Embden–Meyerhof pathway. The genomes of GP72, Pf-5, M18, and A1501 contained genes encoding a fructose-specific IIA component, I-phosphofructokinase. This enzyme is involved in the fructose dissimilation pathway, catalyzing the conversion of fructose to fructose-1,6-diphosphate [45].

PGPR have a variety of genes related to catabolism and transport of plant-derived compounds, such as amino acids, fatty acids, nucleotides, organic acids, carbohydrates, and other exudates [46,47]. Amino acids are one of the major components of root exudates. Accordingly, there were at least 500 genes involved in amino acid transport and metabolism in the genomes of GP72, Pf-5, and M18, and more than 300 in the genome of A1501.

The ability to catabolize aromatic compounds in exudates is one strategy that could confer a selective advantage in the rhizosphere environment. Oxygenases play key roles in the chemical transformation of recalcitrant organic compounds [48,49]. *P*. *putida* modifies diverse aromatics to common intermediates, which feed into central pathways [50]. For example, *P*. *putida* KT2440 is able to use aromatic compounds including benzoate, phenylacetate, tyrosine, and vanillate, as the sole carbon and energy source [51]. There were 21 genes encoding dioxygenases (DOs) in the genome of GP72, 22 in M18, 21 in Pf-5, and 9 in A1501. The DOs in GP72, including benzoate 1,2-dioxygenase, anthranilate 1,2-dioxygenase, protocatechuate 3,4-dioxygenase, and catechol 1,2-dioxygenase, were related to degradation of aromatic compounds. We compared the degradation pathways of aromatic compounds, including the three main pathways (Table 2) and several peripheral pathways, among the four species (Additional file 1).

**Table 2:** Comparison of putative genes related to main pathways for central aromatic catabolism among pseudomonad PGPR

			**Homologs**^**a**^**in PGPR**		
**Gene**	**Product name**	**GP72 ORF ID MOK**_**0**	**Pf**-**5 ORF ID PFL**_	**M18 ORF ID PAM18**_	**A1501 ORF ID PST**_
**3**-**oxoadipate** (**β**-**ketoadipate**) **pathway**					
Catechol degradation III (ortho-cleavage pathway) to 3-oxoadipate enol-lactone					
*catA*	**catechol 1, 2-dioxygenase** [**EC:1.13.11.1**]	1630	3860	2532	1674
*catC*	muconolactone D-isomerase [EC:5.3.3.4]	1631	3861	2531	1673
*catB*	muconate cycloisomerase [EC:5.5.1.1]	1632	3862	2530	1672
*catR*	transcriptional regulator [COG0583]	1633	3863	2529	- ^b^
Protocatechuate degradation II (ortho-cleavage pathway) to 3-oxoadipate enol-lactone					
*pcaG*	**protocatechuate 3**,**4**-**dioxygenase**, **alpha subunit** [**EC**:**1**.**13**.**11**.**3**]	1264	5395	0155	1250
*pcaH*	**protocatechuate 3**,**4**-**dioxygenase**, **beta subunit** [**EC**:**1**.**13**.**11**.**3**]	1265	5396	0154	1249
*pcaH*	**protocatechuate 3**,**4**-**dioxygenase**, **beta subunit** [**EC**:**1**.**13**.**11**.**4**]	1266	5396	0154	1249
*pcaQ*	LysR family transcriptional regulator, pca operon transcriptional activator [KO:K02623]	1267	5397	0153	1248
*pcaH*	**protocatechuate 3**,**4**-**dioxygenase**, **beta subunit** [**EC**:**1**.**13**.**11**.**3**]	2952	1320	-	-
*pcaG*	**protocatechuate 3**,**4**-**dioxygenase**, **alpha subunit** [**EC**:**1**.**13**.**11**.**3**]	2953	1321	-	-
*pcaT*	MFS transporter, MHS family, dicarboxylic acid transporter PcaT [KO:K02625]	2954	1322	0225	-
*pcaB*	3-carboxy-cis,cis-muconate cycloisomerase [EC:5.5.1.2]	2955	1323	0226	1257
*pcaC*	4-carboxymuconolactone decarboxylase [EC:4.1.1.44]	2957	1325	0227	1259
3-Oxoadipate enol-lactone degradation to succinyl-CoA					
*pcaD*	3-oxoadipate enol-lactonase [EC:3.1.1.24]	2956	1324	0226	1258
*pcaI*	Acyl CoA:acetate/3-ketoacid CoA transferase, alpha subunit [EC:2.8.3.12]	2949	1317	0222	1254
*pcaJ*	Acyl CoA:acetate/3-ketoacid CoA transferase, beta subunit [EC:2.8.3.12]	2950	1318	0223	1255
*pcaR*	beta-ketoadipate pathway transcriptional regulators, PcaR/PcaU/PobR family [K02624]	2946	1315	0156	1253
*pcaK*	MFS transporter, AAHS family, 4-hydroxybenzoate transporter [KO:K08195]	2947	1316	0231	-
*pcaK*	MFS transporter, AAHS family, 4-hydroxybenzoate transporter [KO:K08195]	2948	1316	0231	-
*pcaF*	3-oxoadipyl-CoA thiolase [EC:2.3.1.174]	2951	1319	0224	1256
**Homogentisate pathway and catabolism of phenylalanine and tyrosine**					
L-Phenylalanine degradation					
*phhA*	phenylalanine-4-hydroxylase [EC:1.14.16.1]	1525	1611	4167	3562
Tyrosine degradation I to acetoacetate and fumarate					
*tyrB*	aromatic-amino-acid transaminase [EC:2.6.1.57]	1527	1609	4169	3564
*tyrB*	aromatic-amino-acid transaminase [EC:2.6.1.57]	3418	2045	1824	2998
*hppD*	**4**-**hydroxyphenylpyruvate dioxygenase** [**EC**:**1**.**13**.**11**.**27**]	5394	3387	-	-
*hppD*	**4**-**hydroxyphenylpyruvate dioxygenase** [**EC**:**1**.**13**.**11**.**27**]	1257	5385	0238	0200
*hmgA*	**homogentisate 1**,**2**-**dioxygenase** [**EC**:**1**.**13**.**11**.**5**]	2407	0967	3036	-
*hmgC*	maleylacetoacetate isomerase [EC:5.2.1.2]	2409	0969	3038	-
*hmgB*	fumarylacetoacetase [EC:3.7.1.2]	2408	0968	3037	-
Tyrosine (4-Hydroxyphenylacetate/3-Hydroxyphenylacetate) degradation II to succinate					
*hpaC*	4-hydroxyphenylacetate-3-hydroxylase small chain [EC:1.14.13.3]	5674	3357	0848	-
*hpaB*	4-hydroxyphenylacetate-3-hydroxylase large chain [EC:1.14.13.3]	5675	3356	0849	-
*hpaD*	**3**,**4**-**dihydroxyphenylacetate 2**,**3**-**dioxygenase** [**EC**:**1**.**13**.**11**.**15**]	5409	3373	0815	-
*hpaE*	5-carboxymethyl-2-hydroxymuconic-semialdehyde dehydrogenase [EC:1.2.1.60]	5410	3372	0816	-
*hpaF*	5-carboxymethyl-2-hydroxymuconate isomerase [EC:5.3.3.10]	5528	1486	-	-
*hpaF*	5-carboxymethyl-2-hydroxymuconate isomerase [EC:5.3.3.10]	5408	3374	0814	-
*hpaG*	5-oxopent-3-ene-1,2,5-tricarboxylate decarboxylase, C-terminal subunit [EC:4.1.1.68]	5411	3371	0817	-
*hpaG*	5-oxopent-3-ene-1,2,5-tricarboxylate decarboxylase, N-terminal subunit [EC:4.1.1.68]	5412	3370	0818	-
*hpaA*	4-hydroxyphenylacetate catabolism regulatory protein [KO:K02508]	5413	3369	0819	-
*hpaH*	2-oxo-hept-3-ene-1,7-dioate hydratase [EC:4.2.1.-]	5406	3376	0812	-
*hpaI*	2,4-dihydroxyhept-2-ene-1,7-dioic acid aldolase [EC:4.1.2.-]	5405	3377	0811	-
*hpaI*	2,4-dihydroxyhept-2-ene-1,7-dioic acid aldolase [EC:4.1.2.-]	2721	-	-	-
*gabD*	succinate-semialdehyde dehydrogenase (NADP+) [EC:1.2.1.16]	5324	0185	0260	0096
*gabD*	succinate-semialdehyde dehydrogenase (NADP+) [EC:1.2.1.16]	2687	0185	-	0740
Phenylethylamine degradation II to phenylacetate					
*peaD*	quinohemoprotein amine dehydrogenase, beta subunit [TIGR03907]	1726	4117	-	-
*peaC*	quinohemoprotein amine dehydrogenase, gamma subunit [pfam08992]	1727	4118	-	-
*peaA*	quinohemoprotein amine dehydrogenase, alpha subunit [TIGR03908]	1729	4120	-	-
*peaE*	phenylacetaldehyde dehydrogenase [EC:1.2.1.39]	1734	4130	-	-
*peaE*	phenylacetaldehyde dehydrogenase [EC:1.2.1.39]	5496	3217	0867	-
**Phenylacetyl**-**CoA pathway**					
*paaF*	phenylacetate-CoA ligase [EC:6.2.1.30]	0341	3132	-	-
*paaD*	acyl-CoA thioesterase [EC:3.1.2.-]	0343	3131	-	-
*paaG*	phenylacetate-CoA oxygenase, PaaG subunit [KO:K02609]	0340	3133	-	-
*paaH*	phenylacetate-CoA oxygenase, PaaH subunit [KO:K02610]	0339	3134	-	-
*paaI*	phenylacetate-CoA oxygenase, PaaI subunit [KO:K02611]	0338	3135	-	-
*paaJ*	phenylacetate-CoA oxygenase, PaaJ subunit [KO:K02612]	0337	3136	-	-
*paaK*	phenylacetate-CoA oxygenase, PaaK subunit [KO:K02613]	0336	3137	-	-
*paaN*	MaoC_dehydratas/NAD-dependent aldehyde dehydrogenases [KO:K02618]	0332	3140	-	-
*paaE*	3-oxoadipyl-CoA thiolase [EC:2.3.1.16]	0342	1319	0224	1256
*paaB*	enoyl-CoA hydratase [EC:4.2.1.17]	0345	3130	-	-
*paaA*	enoyl-CoA hydratase [EC:4.2.1.17]	0346	-	-	-
*paaC*	3-hydroxybutyryl-CoA dehydrogenase [EC:1.1.1.157]	0344	-	-	-
*paaY*	phenylacetic acid degradation protein PaaY [KO:K08279]	0347	3129	-	-
*paaX*	phenylacetic acid degradation operon negative regulatory protein PaaX [KO:K02616]	0348	3128	-	-

^a^ Homologous genes were analyzed at 60% identity threshold using IMG software.

^b^ “-” No homologs were present in the compared genome.

Genes encoding components of the 3-oxoadipate pathway, which is common in soil and plant-associated microorganisms [52], were present in the genomes of all four PGPR analyzed in this study. The pathway has two branches: one converting catechol and the other converting protocatechuate. Both branches produce two tricarboxylic acid cycle intermediates. Based on the comparative genomic analysis, the former branch may derive from the degradation of tryptophan [53], benzoate [54], salicylate [55], phenol [56,57], and so on, while the protocatechuate branch is generated from 4-hydroxybenzoate [58], and numerous lignin monomers such as vanillate [59] and quinate [60]. Analyses of aromatic compound catabolism not only reveal the broad metabolic activities of PGPR, but also provide insights into mediating the production of useful secondary metabolites such as phenazine [61], pyocyanin (PYO) [62], and C-1027 [63].

Some bacteria and fungi degrade tyrosine (Tyr) via the central intermediate homogentisate (2,5-dihydroxyphenylacetate). The reaction proceeds with conversion of Tyr into 4-hydroxyphenylpyruvate (HPP) (by tyrosine aminotransferase), and then formation of homogentisate (by HPP dioxygenase), which is degraded via the homogentisate central pathway [64]. The central pathway involves three enzymes: homogentisate 1,2-dioxygenase (HmgA), fumarylacetoacetate hydrolase (HmgB), and maleylacetoacetate isomerase (HmgC), and yields fumarate and acetoacetate [65]. The comparative genomic analysis indicated that GP72, Pf-5, and M18 could degrade Tyr via the homogentisate pathway. Some Gram-positive bacteria can convert Tyr into homoprotocatechuate (3,4-dihydroxyphenylacetate), rather than homogentisate, producing pyruvate and succinate [66,67]. A variety of microorganisms contain 3,4-dihydroxyphenylacetate 2,3-dioxygenases (HPCD) [68,69], such as Fe(II) (HPCD) and Mn(II) (MndD). These enzymes contain different active sites resulting in different structures and HPCD activities [70,71]. The annotated amino acid sequence of the HPCD from GP72 exhibited 63–64% identities with those of the corresponding enzymes from *Escherichia coli*[68] and *Klebsiella pneumonia*[69]. Further research is required to characterize the activities of HPCD in different *Pseudomonas* species.

In a few organisms, phenylethylamine, an intermediate of phenylalanine degradation, can be converted into phenylacetaldehyde by quinohemoprotein amine dehydrogenase, and then transformed into phenylacetate by phenylacetaldehyde dehydrogenase [72-74]. The corresponding genes were predicted in the genomes of GP72 and Pf-5, but they were not located in a single operon.

Phenylacetyl-CoA is derived from various substrates such as phenylalanine, lignin-related aromatic compounds, and environmental contaminants, and can be degraded to succinyl-CoA and acetyl-CoA [75,76]. Based on the genomic comparison at the 60% identity threshold, we found that the phenylacetate degradation pathway was present in GP72 and Pf-5. However, this pathway was not detected in M18 or A1501 at the same identity level, indicating potentially different evolutionary directions in specific niches.

Five putative phenylpropionate dioxygenases and related ring-hydroxylating dioxygenases of unknown specificity can also participate in aromatic compound catabolism [77].

Plant-derived substances not only serve as important carbon and energy sources for rhizosphere bacteria, but also influence bacterial behaviors [78,79]. For example, the ratio of rhizospheric carbon:nitrogen (C:N) can alter the nutritional status of *Rhizoctonia solani*, making the fungus a pathogen [80]. Tomato root exudates promote germination of spores of the tomato root pathogen *F*. *oxysporum* f. sp. *radicis*-*lycopersici*, whereas the biocontrol agent *P*. *fluorescens* WCS365 delays this process [81]. Microarray analyses showed that root exudates affected the transcriptome of *P*. *aeruginosa* PAO1 by influencing genes encoding enzymes related to alginate biosynthesis and twitching motility [82]. Therefore, the production of plant-derived exudates could alter the composition of rhizospheric microorganism communities. Further research is required to investigate the molecular mechanisms underlying changes in community structure.

#### Transport

Consistent with the abundance of genes related to metabolism of plant-derived substances, the four PGPR contained many putative transport genes related to substrate uptake and excretion (Table 3). GP72 and Pf-5 contained similar numbers of transport genes. In bacteria, secretion systems play an important role in transport or translocation of effectors for adaptation to their natural surroundings. The genomes of these four PGPR contained type I, type II, type IV, type V, and type VI secretion systems, as well as the chaperone-usher secretion system and the twin-arginine translocation system. M18 also contained the Type III (flagellar/pathogenesis) secretion system, a key virulence factor in pathogenic *Pseudomonas*[83].

**Table 3:** Numbers of putative genes encoding transporters in genomes of four pseudomonad PGPR

	**GP72**	**Pf-5**	**M18**	**A1501**
**Carbohydrate transporter genes**	**125**	**108**	**103**	**46**
Major facilitator family (MFS)	76	75	75	17
ATP binding cassette (ABC) family	32	19	11	11
Tripartite ATP-Independent periplasmic transporter family	5	5	10	14
Phosphotransferase system (PTS)	6	6	4	3
Gluconate transporter GntT	6	5	3	1
**Amino acid transporter genes**	**181**	**207**	**109**	**71**
ABC transporter	137	156	70	55
Lysine exporter (LysE) family	18	24	13	11
Amino acid-polyamine-organocation (APC) family	16	21	21	5
Drug/metabolite transporter (DMT) family	10	6	5	0
**Transporter genes related to defense**	**79**	**78**	**48**	**38**
ABC transporter	37	46	24	19
Resistance-nodulation-cell-division (RND) family	35	25	15	15
Multidrug and toxic compound extrusion (MATE) family	3	4	2	2
Small multidrug resistance (SMR) family	4	3	7	2

#### Defense pathways

Previous studies showed that GP72 resists streptomycin up to a concentration of 100 μg ml^-1^, and tolerates salt (5% NaCl solution). Both of these resistances are stronger than those of *P*. *chlororaphis* strain 30–84 [7]. Antibiotic resistance assays showed that GP72 displays resistance to penicillin, spectinomycin, streptomycin, and tetracycline. Here, our genomic analyses confirmed different kinds of defenses in the four PGPR, including resistance/tolerance to heavy metals, temperature stress, osmotic stress, oxidative stress, and multiple drugs.

Many essential trace elements contain metal ions that are important components of the active sites of many enzymes. As such, they play a vital role in many biological processes, including photosynthetic and respiratory pathways. However, most heavy metals are toxic at higher concentrations. For example, copper ions can damage the cytoplasmic membrane of *E*. *coli* by catalyzing harmful redox reactions [84]. In many regions, agricultural soils are heavily contaminated with various heavy metals originating from chemical fertilizers and industrial processes. Consequently, certain soil bacteria have developed resistance to toxic metals, either via active efflux mechanisms to pump the toxic metals out [85], or by enzymatic detoxification to convert a toxic ion into a harmless one [86,87]. Our genomic analysis revealed many genes related to heavy metal resistance (summarized in Table 4).

**Table 4:** Summary and comparison of putative genes related to metal resistance in four pseudomonad PGPR genomes

			**Homologs**^**a**^**in PGPR**		
**Gene**	**Product name**	**GP72 ORF ID MOK**_**0**	**Pf**-**5 ORF ID PFL**_	**M18 ORF ID PAM18**_	**A1501 ORF ID PST**_
**Copper resistance**					
*copG*	predicted metal-binding protein	0288	2891	4821	3385
*copD*	putative copper export protein	0289	- ^b^	-	-
*copC*	uncharacterized protein, homolog of Cu resistance protein CopC	0290	-	-	-
*copB*	uncharacterized protein involved in copper resistance	0291	2892	2980	3381
*copA*	copper-resistance protein, CopA family	0292	2893	2979	3383
-	uncharacterized copper-binding protein	3441	1966	2156	-
*copR*	heavy metal response regulator	3442	1965	2154	2712
*copS*	heavy metal sensor kinase[EC:2.7.13.3]	3443	1964	2153	-
*copC*	uncharacterized protein, homolog of Cu resistance protein CopC	3597	2543	-	-
*copD*	putative copper export protein	3598	2542	-	-
*cueO*	putative multicopper oxidases	0816	4929	1176	3006
*cueR*	Cu(I)-responsive transcriptional regulator	4686	0709	4886	3614
*copA*	copper-(or silver)-translocating P-type ATPase[EC:3.6.3.4]	4687	0710	1020	3613
*copZ*	copper chaperone	4689	0712	1410	-
**Copper**/**silver resistance**					
*cusR*	two-component system, OmpR family, copper resistance phosphate regulon response regulator CusR	0912	5050	3694	-
*cusS*	two-component system, OmpR family, heavy metal sensor histidine kinase CusS [EC:2.7.13.3]	0913	5051	-	-
*cusC*	heavy metal RND efflux outer membrane protein, CzcC family	0016	-	-	-
*cusB*	heavy metal RND efflux outer membrane protein, CzcC family	0017	-	-	-
*cusB*	Cu(I)/Ag(I) efflux system membrane protein CusB	0018	-	-	2082
*cusA*	Cu(I)/Ag(I) efflux system membrane protein CusA	0019	-	-	2083
*cusF*	Cu(I)/Ag(I) efflux system periplasmic protein CusF	0020	-	-	-
**Arsenic resistance**					
*arsR*	predicted transcriptional regulators	0160	-	2763	2096
*arsB*	arsenical pump membrane protein	0161	2185	2762	-
*arsC*-*2*	arsenate reductase	5909	2184	2761	-
*arsH*	arsenical resistance protein ArsH	5910	2183	2760	2097
*arsC*-*1*	arsenate reductase (glutaredoxin)[EC:1.20.4.1]	5052	4456	4088	2824
**Cobalt**/**zinc**/**cadmium resistance**					
*czcA*	heavy metal efflux pump (cobalt-zinc-cadmium)	1084	5218	2519	3425
*czcB*	RND family efflux transporter, MFP subunit	1085	5219	2518	-
*czcC*	outer membrane protein	1086	5220	-	-
*czcR*	heavy metal response regulator	1087	5221	2516	3421
*czcD*	cation diffusion facilitator family transporter	1088	5222	0397	-
**Chromate resistance**					
*chrA*	chromate transporter, chromate ion transporter (CHR) family	0320	3149	4378	-
*chrB*	uncharacterized conserved protein	4261	-	-	2920
*chrA*	chromate transporter, chromate ion transporter (CHR) family	4262	-	-	2921

^a^ Homologous genes were analyzed at 60% identity threshold using IMG software.

^b^ “-” No homologs were present in the compared genome.

The four pseudomonad PGPR studied contained at least two different copper resistance systems, which resemble those identified in the plant growth-promoting endophytic bacterium *P*. *putida* W619 [15]. One system is periplasmic detoxification encoded by *copABCDGcopRS*, which is well-characterized in the plasmid pPT23D from *P*. *syringae* pv. *tomato* strain PT23.2 [88]. This system is also widely distributed in other *Pseudomonas* species [15,88,89]. Another copper resistance system is the cytoplasmic detoxification system *cue*, which maintains a strict quota of cellular copper in other organisms [90,91]. The Cu(I)-responsive transcriptional regulator CueR [91] activates expression of a copper-translocating P-type ATPase (CopA) [92], a periplasmic multicopper oxidase (CueO) [93], and a copper chaperone (CopZ) [90] under mild copper stress. CopA exports Cu(I) from the cytoplasm to the periplasm, and then Cu(I) is converted into the less toxic Cu(II) form by CueO. A third copper resistance strategy in the genome of GP72 consisted of *cusFABBC* (MOK_00020-00016) and *copRS* (MOK_00012-00013). The *cus* operon is related to periplasmic detoxification, and is exclusively found in Gram-negative bacteria [94,95]. Therefore, the mechanism for copper resistance in *P*. *chlororaphis* is very complex, and has not been completely characterized yet. Further research is required to clarify the details of this system.

*Pseudomonas* spp. have arsenic-resistance genes (*arsRB*, *arsCH* and *arsC*) that are dispersed throughout the genome. The chromosomal *ars* operon was characterized in *P*. *aeruginosa*. A homologous *ars* operon was detected in some, but not all, *Pseudomonas* species, indicating that some other mechanisms are involved in arsenic resistance in pseudomonads [96]. Our genomic analysis indicated that the GP72 genome lacked a homologous gene encoding an arsenite- and antimonite-stimulated ATPase (ArsA). However, a previous study showed that ArsB could export arsenite ions in the absence of ArsA in *E*. *coli*[97]. Since ArsB was predicted in the genome of GP72, we can assume that this strain also shows arsenic resistance. The *czcABCRD* operon encoding a cation-proton antiporter, which is responsible for cobalt, zinc, and cadmium resistances [98], was predicted in the genomes of GP72 and Pf-5. Other genes found in their genomes may also be related to heavy metal resistance, such as homologs of *chrA* and *chrB* genes involved in chromate resistance [99], and homologs of genes encoding siderophores that participate in metal homeostasis in *P*. *aeruginosa*[100].

In recent years, multidrug resistance has reached alarming levels, especially in the field of medicine [101]. Such resistance mechanisms have been fully described by Alekshun and Levy [102]. Some PGPR strains contain a broad spectrum of putative multidrug resistance genes, including genes related to well-developed efflux systems [103], penicillin-binding protein-mediated resistance [104], and enzymes that degrade antibiotics (Additional file 2). Efflux systems contribute significantly to resistance to multiple antimicrobial compounds. This is a very important mechanism to enhance biological fitness [105]. Like other *Pseudomonas* species, GP72 contained 37 putative ABC transporters, which potentially participate in the uptake or efflux of toxic metabolites and other drugs. Some secondary transport system genes were also present in GP72 (Table 3); there were 35 genes encoding RND family members, three genes for MATE family members, and four genes for SMR family members. All pseudomonad PGPR contained genes encoding the efflux pumps TtgABC and TtgDEF (toluene tolerance genes). These enzymes prevent the accumulation of toluene and other related aromatics, such as phenol [106]. Genes encoding an MexEF-OprN efflux pump, a member of the RND family, were also present in the genomes of GP72, Pf-5, M18, and A1501, but the order of the efflux pump genes in the genome differed among the four strains. The efflux pump operon is upregulated by MexT under nitrosative stress and chloramphenicol stress [107]. Overexpression of this system can decrease the production of several secondary metabolites such as PYO, elastase, and rhamnolipids [108]. AcrB (homologous to MOK_00261 in the GP72 genome), which also belongs to the RND family [109], plays a role in pumping out basic dyes (such as acriflavine), most antibiotics (except aminoglycosides), and detergents (such as bile salts, Triton X-100, and SDS) [110]. In conclusion, the genomic data indicated that these PGPR harbor genes that can confer resistance to multiple drugs, including penicillin, aminoglycosides, fluoroquinolones, trimethoprim-sulfamethoxazole, lipid A, and acriflavine.

**Figure 6:**
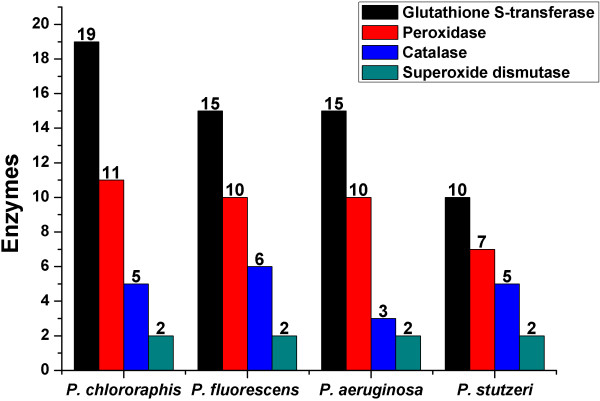
**Numbers of predicted enzymes with roles in oxidative stress response.** Predicted proteins with roles in the oxidative stress response found in *P*. *chlororaphis* GP72, *P*. *fluorescens* Pf-5, *P*. *aeruginosa* M18, and *P*. *stutzeri* A1501. Four types of enzymes (glutathione S-transferase, peroxidase, catalase, and superoxide dismutase) were compared among the four species.

Bacteria that inhabit the rhizosphere of plants can use plant-derived compounds as nutrients; however, they must be able to tolerate damaging compounds produced by plants, such as reactive oxygen species (ROS). Several ROS are continuously produced during aerobic metabolism of plants. They participate in regulating plant cell expansion [111] and other biological processes. ROS show antimicrobial activities [112], as they can damage proteins, nucleic acids, and cell membranes. Rhizospheric bacteria produce several enzymes to resist oxidative stress [113]. Genes encoding these enzymes have already been identified in the genomes of Pf-5 and A1501 [17,18]. Putative ROS-detoxifying enzymes in GP72 included 11 peroxidases, five catalases, two superoxide dismutases, and 19 glutathione S-transferases. There was no significant difference in the numbers of these enzymes among the four PGPR (Figure 6). Genes encoding regulators of the oxidative stress response, including the two-component regulator GacS/GacA [114], SoxR, and OxyR [113,115] were present in the genomes of GP72, Pf-5, M18, and A1501. However, a homolog of SoxR in *P*. *aeruginosa* did not function as a key regulatory player in the bacterial oxidative stress response [116]. Exopolysaccharides such as alginate [117] and polyhydroxyalkanoates (PHAs) [118] are important for tolerance to oxidative stress under ambient pressure. For instance, PHA accumulation enhances the survival of pseudomonads under salinity stress, oxidative stress, and cold-shock [119,120]. Additionally, a pyrroloquinoline-quinine (PQQ) synthase expressed in *E*. *coli* improves its resistance to photodynamically produced ROS [121].

Rhizosphere bacteria usually survive in a changeable environment; therefore, they have evolved several traits related to adaptation [122]. The genomes of GP72, Pf-5, M18, and A1501 contained homologs of genes related to tolerating cold-shock, including *cspACDG*, which is constitutively expressed at 37°C [123]. In *P*. *aeruginosa* cells, a temperature increase from 30 to 45°C enhances production of 17 proteins, including the heat-shock proteins DnaK and GroEL [124]. A chaperone system formed by DnaK, DnaJ, and GrpE proteins modulates the heat-shock response in *E*. *coli*[125] (Additional file 2). As an opportunistic pathogen, *P*. *aeruginosa* has evolved to survive in diverse stressful environments. A microarray analysis showed that *P*. *aeruginosa* synthesizes osmoprotective compounds, such as hydrophilins and osmoprotectants, to cope with osmotic stress [126,127]. Glycine betaine (GB), a major osmoprotectant for many bacteria [128], can accumulate via *de novo* synthesis or via absorption from the environment [126]. Mutant analyses and ^13^C NMR studies confirmed GB catabolism in *P*. *aeruginosa*[129]. Previously, it was shown that GP72 shows strong osmotic stress tolerance [7]. The genomic analysis in this study showed that the genomes of GP72, Pf-5, and M18 contained at least one complete gene set required for conversion of GB to glycine; this gene set included *gbcAB*, *dgcAB*, and *soxGADB*. In contrast, A1501 contained only a homolog of the *betAB* operon, which encodes a system for oxidation of choline to GB under osmotic stress conditions [130]. Osmoregulated periplasmic glucans are highly branched oligosaccharides found in the periplasm of Gram-negative bacteria. They are probably produced in response to periplasmic osmolality, which is controlled by the products of *mdoD* and *mdoG*[131]. Enteric bacteria can modulate their cytoplasmic osmolality through mobilizing K^+^, glutamate, and other compatible solutes, such as trehalose, proline, and GB [132]. K^+^ first responds to osmotic upshifts via the transporters Trk and Kdp, possibly acting as a putative osmoregulatory second messenger [126,133]. The genes related to osmotic stress tolerance are listed in Additional file 2.

We found that resistance genes were present in the genomes of all four PGPR, although some genes showed low similarity to others. These results indicated that PGPR may undergo long-term evolution to adapt to specific ecological niches. To adapt to changeable environments, each pseudomonad PGPR strain has a complex array of regulatory networks, including sigma factors, transcriptional regulators, and a variety of two-component transcriptional regulators.

### Rhizosphere colonization

A confocal laser scanning microscopy analysis showed that *P*. *fluorescens* WCS365 and *P*. *chlororaphis* PCL1391 are able to effectively colonize the tomato rhizosphere [134], and the major traits for niche competition were identified [135]. Development of new genetic approaches such as in vivo expression technology (IVET) together with “omic” technologies has provided opportunities to identify genes required for rhizosphere competence, and to elucidate the genetic mechanisms of plant–microbe interactions [14,136]. Pseudomonad PGPR show certain competitive colonization traits, such as motility and the ability to attach to the root surface.

First, motility is a major trait for the competitive tomato root-tip colonization of *P*. *fluorescens*, based on chemotaxis [137,138]. We found genes related to chemotaxis and motility in the genomes of the four PGPR; GP72 contained 14 genes responsible for various aspects of chemotaxis, including genes encoding a two-component system (CheA/CheY). The activity of the histidine kinase CheA can be regulated by methyl-accepting chemoreceptor proteins (MCPs) during chemotaxis [139]. Swimming behavior can be initiated when the phosphorylated CheY binds to the flagellar switch protein, Flim [140]. In the present study, we found 28 genes encoding MCPs and 40 genes associated with flagella biosynthesis, including the *flg* and *fli* operons.

The second trait of competitive colonization is attachment to the root surface. In this study, several genes involved in attachment were predicted in the PGPR genomes (Additional file 3). The functions of some genes have been confirmed experimentally in certain *Pseudomonas* species, including genes associated with type IV pili and twitching motility [141], genes for biosynthesis of alginate [142], hemolysin [135,143], filamentous hemagglutinin [144], and lipopolysaccharide O-antigen [145], and genes for other enzymes or factors involved in adhesion [135]. For instance, twitching motility, a type of flagella-independent surface motility mediated by type IV pili, is a mechanism of rapid bacterial colonization [141]. As well as the common type IV pilus assembly proteins, we identified a second set of genes in *P*. *chlororaphis* GP72 that were previously reported to play roles in the biogenesis of the Flp subfamily of type IVb tight adherence (Tad) pili [146]. However, *tad* genes were not found in the genomes of the other three PGPR at the 60% identity threshold, when compared with the genome of GP72. Tad pili are an essential and conserved host-colonization factor in *Bifidobacterium* species [147]. Therefore, we can speculate that the *tad* genes are probably derived from organisms outside of the genus *Pseudomonas*. In strain *P*. *putida* KT2440, a series of *rap* genes (*root*-*activated promoters*) were identified during maize root colonization by IVET [148]. Some of the promoters isolated by *rap* fusions responsible for adhesion were present in the genomes of GP72, Pf-5, and M18, such as *secB* (*rap*_*1*-*2*_ fusion) [135] and *algD* (*rap*_*2*-*45*_ fusion) [142]. The genetic locus *aggA*, which is involved in agglutination and adherence [149], was also predicted in the genomes of GP72 and Pf-5. Espinosa-Urgel et al. [143] characterized several *mus* (*mutants unattached to seeds*) loci in *P*. *putida*, and confirmed that mutants of these loci show impaired attachment to corn seeds. The genome of GP72 contained four *mus* loci: *mus*-*13*, *mus*-*21*, *mus*-*24*, and *mus*-*27*, with possible functions as a carbon starvation protein, transporter, calcium-binding protein, and hemolysin, respectively. Genes involved in competitive rhizosphere colonization have been well studied in *P*. *fluorescens*. These include *xerC*, which encodes a site-specific recombinase. *xerC* is a homolog of *sss* in *P*. *chlororaphis* PCL1391; *sss* plays a role in phase variation caused by DNA rearrangements [3,150]. The *nuo* operon encodes subunits of NADH: ubiquinone oxidoreductase, which is related to ATP-dependent rotation of flagella [145]. Some of the genes isolated by IVET and identified to play roles in plant–microbe interactions [14,151] were present in the genomes of GP72, Pf-5, M18, and A1501 when compared with the genome of *P*. *fluorescens* SBW25 (data not shown). However, it remains to be confirmed whether these genes specifically contribute to rhizosphere competence.

GP72, Pf-5, and A1501 lacked virulence factors found in plant pathogens, such as the type III secretion system, phytotoxins, and exoenzymes associated with cell wall degradation. Homologs of genes encoding phytotoxins produced by *P*. *syringae* (coronatine, syringomycin, syringopeptin, tabtoxin, and phaseolotoxin) [152] were also absent from the genomes of GP72, Pf-5, M18, and A1501. Their genomes did not contain genes related to the biosyntheses of cellulases, pectinases, or pectin lyases, which play roles in the degradation of cell wall components. Therefore, the lack of these genes can result in efficient rhizosphere colonization and improvement of plant growth.

### Biocontrol activities

Biocontrol activities are important mechanisms by which PGPR suppress plant pathogens. The main biocontrol strategy is the production of a spectrum of antibiotics [2]. The antibiotics produced by the biocontrol agents GP72, Pf-5, and M18 are listed in Table 5. Phenazines are versatile secondary metabolites produced by *P*. *fluorescens*, *P*. *chlororaphis*, and *Pseudomonas aureofaciens*[153]. These compounds play critical roles in the biological control activities of *Pseudomonas* spp. [5]. Previous studies showed that GP72 can completely suppress various phytopathogens, mainly because of the production of PCA and 2-OH-PHZ. Clusters of phenazine-compound biosynthetic genes were present in the genomes of both GP72 and M18, but the genes differed between the two species. The GP72 genome contained *phzO*, encoding an aromatic monooxygenase [154] that converts PCA to 2-OH-PHZ, whereas M18 contained two *phz* gene clusters and one set of modified *phzMS* genes. *phzM* and *phzS* encode a putative S-adenosylmethionine-dependent N-methyltransferase and a putative flavin-dependent hydroxylase, respectively. They participate in the conversion of PCA to PYO in *P*. *aeruginosa*. PYO is a virulence factor to cystic fibrosis patients infected by pathogenic pseudomonads [155]. However, M18 does not produce detectable levels of PYO at 28°C, mainly because of the temperature-dependent expression of *phzM* and its regulatory genes *lasI* and *ptsP*. The biocontrol activity of M18 is, therefore, not attributed to PYO but to PCA [33], and it shows lower pathogenicity than other closely related strains. Previous studies showed that some plant pathogens are more strongly inhibited by 2-OH-PHZ than by PCA [154]. Another important antibiotic is Plt, which is produced by both Pf-5 and M18. Strains with the ability to produce the insect toxin ‘Fit’ (*P*. *fluorescens*insecticidal toxin) [156] show potent insecticidal activity [157]. The *fitD* gene encoding the cytotoxin in GP72 showed 84% identity to that in Pf-5. The amino acid sequence of Fit shared 77% amino acid identity with the insect toxin Mcf (makes caterpillars floppy) produced by the entomopathogen *Photorhabdus luminescens*[157].

**Table 5:** Secondary metabolites produced by pseudomonad biocontrol strains

	**GP72**	**Pf-5**	**M18**
Phenazine	PCA, 2-OH-PCA	- ^a^	PCA, PYO ^b^
Pyoluteorin (Plt)	-	Plt	Plt
Pyrrolnitrin (Prn)	Prn	Prn	-
2,4-diacetylphloroglucinol (DAPG)	-	DAPG	-
Hydrogen cyanide (HCN)	HCN	HCN	HCN
*P*. *fluorescens*insecticidal toxin (Fit)	Fit	Mcf	-
Pyoverdine (Pvd)	Pvd	Pvd	Pvd
Pyochelin (Pch)	-	Pch	Pch
Achromobactin (Acr)	Acr	-	-

^a^ “-” Secondary metabolite is absent from that strain, based on previous studies and genomic sequence information.

^b^ M18 did not produce detectable levels of PYO at 28°C [33].

Fluorescent pseudomonads can produce pyoverdin (Pvd), a fluorescent siderophore, and chelate Fe(III) efficiently under low-iron conditions to improve their biocontrol activity [158,159]. The fluorescent pseudomonads GP72, Pf-5, and M18 contained the complete Pvd biosynthetic gene cluster. In addition, Pf-5 and M18 contained genes encoding another siderophore, Pch, which has antifungal activity [160]. GP72 lacked these genes, but it contained putative genes for synthesis of achromobactin (Acr), a temperature-regulated secondary siderophore. The related biosynthetic gene clusters in GP72 included *acsFDECBA*, *yhcA*, and *acrABCD*, which are responsible for the biosynthesis of Acr, permease, and a specific outer membrane receptor, respectively [161,162]. Siderophores can bind metals other than iron [163] and, therefore, can play roles in sequestering toxic metals including aluminum, cobalt, copper, and lead [100]. GP72 contained a locus (MOK_02694) encoding a nickel-uptake substrate-specific transmembrane protein, adjacent to the *acr* operon. Acr in GP72 may be involved in metal transport, signaling pathways, or antimicrobial activities. The comparative genomic analysis indicated that there was no homology of the *acr* operon between Pf-5 and M18. As well as producing their own siderophores, *Pseudomonas* can also use siderophores produced by other microorganisms. For example, A1501 may obtain iron via heterologous siderophores, since it lacks pathways for siderophore biosynthesis [18]. Genes involved in the uptake of soluble Fe(III) complexes, that is, those encoding putative outer membrane receptors, were present in the genomes of the four pseudomonads: 31 genes in GP72, 45 in Pf-5, 36 in M18, and 24 in A1501. The variable iron acquisition systems among *Pseudomonas* reflect their large capacity for niche colonization, providing insights into how their biocontrol abilities can be improved. Therefore, the availability of complete genome sequences provides an excellent opportunity to explore the diversity and evolution of biosynthetic pathways in different species/strains [153,164].

### Direct plant-growth promotion

Rhizobacteria can directly promote plant growth, and some strains have been developed as ‘biofertilizers’. The mechanisms underlying plant-growth promotion include nitrogen fixation, increased nutrient availability, production of phytohormones, and so on [165]. The biofertilizers *Azotobacter*[12] and *P*. *stutzeri*[18], both of which belong to the Pseudomonadaceae, are able to fix nitrogen. The genome of A1501 contains a cluster of 59 genes specific to nitrogen fixation, and the *nif* operon shows a high degree of similarity to that in the genome of *Azotobacter vinelandii*. Therefore, we compared A1501 with GP72, Pf-5, and M18 at a threshold of 30% identity to screen for putative genes related to nitrogen fixation. The analyses revealed 13, 13, and 14 homologous genes in GP72, Pf-5, and M18, respectively (Additional file 4); however, these three strains lacked the nitrogenase complex-encoding genes *nifDK*[166,167]. We conducted a similar screen for denitrification genes; of 45 genes in A1501, 7 homologs were found in the genome of Pf-5, and 21 in the genome of GP72. We can speculate that the low identities may be because of the relatively distant evolutionary relationship, as shown in the phylogenetic analysis (Figure 4). GP72 and M18 contained several genes involved in denitrification: *narL* and *narX*, which encode a two-component regulatory system; *narGHJI*, which encodes respiratory nitrate reductase [168]; and *nor* genes, which are involved in nitric oxide metabolism. Previous studies reported that *P*. *fluorescens* and *P*. *chlororaphis* produce N_2_O as the only detectable gaseous product of denitrification [169], while *P*. *stutzeri* emits only N_2_, and *P*. *aeruginosa* produces both N_2_ and N_2_O [170]. Thus, the denitrification process can accommodate large quantities of anthropogenic nutrients, converting nitrate into nitrogen. This could decrease nitrate accumulation and counteract eutrophication in the environment [171].

Limited quantities of soluble phosphate can restrict plant growth. The genomes of GP72, Pf-5, M18, and A1501 contained several genes encoding nonspecific phosphatases, inositol phosphate phosphatases, and C-P lyases. These enzymes catalyze the conversion of insoluble phosphorus into plant-available forms, thereby facilitating plant growth [172]. In addition, many PGPR can produce phytohormones to stimulate plant growth. GP72 can synthesize IAA [7] via a tryptophan-dependent pathway [173], since it contained genes encoding tryptophan-2-monooxygenase (*iaaM*, MOK_03651/04103/05943) and indoleacetamide hydrolase (*iaaH*, MOK_00889/01660/02975). A1501 lacks the putative IAA synthesis pathway [18]. The genomes of GP72, Pf-5, M18, and A1501 contained putative 1-aminocyclopropane-1-carboxylate (ACC) deaminases. This enzyme can counteract the ethylene response in plants by degrading the ethylene precursor ACC. In other *Pseudomonas* strains, ACC deaminases promote root elongation and suppress plant diseases [174]. Biosynthetic genes for PQQ, a plant-growth promotion factor, are clustered in the conserved *pqqABCDEF* operon [175]. This operon was present in the genomes of Pf-5, M18, and A1501. GP72 lacked *pqqA*, but the enzyme encoded by *pqqA* is not required for biosynthesis of PQQ in *Methylobacterium*[176].

## Conclusions

We analyzed plant growth-promoting traits by a comparative genomics analysis of four representative pseudomonad PGPR strains. The genes that were conserved among the different *Pseudomonas* species have provided clues to the common characteristics of pseudomonad PGPR, such as rhizosphere competence traits (nutrient catabolism and transport, resistance to various environmental stresses, and rhizosphere colonization). The strain-specific genes differentiated each strain on the basis of its lifestyle, specific ecological adaptations, and physiological role in the rhizosphere. The recently reported genome of *P*. *chlororaphis*, together with other sequenced strains of different species of pseudomonad PGPR, provides insights into the genetic basis of diversity and adaptation to specific environmental niches. Comparative genomic analyses, combined with certain IVET-based analyses, can reveal many genetic factors related to plant growth promotion. First, the strong adaptability of PGPR to their environment is related to putative genes involved in catabolism and transport of plant-derived compounds and resistance to various environmental stresses (heavy metals, ROS, cold-, heat-, or osmotic-shock, and multiple drugs). These genes were very common in the genomes of PGPR, especially those of *P*. *chlororaphis* and *P*. *fluorescens*, and provide the foundation for rhizosphere fitness. Second, we compared genes involved in rhizosphere colonization. Some related genes showed low similarity between *P*. *chlororaphis* GP72 and the other three strains, including biosynthetic genes for the O-antigen and type IV pilus assembly. Hence, GP72 may have stronger rhizosphere competence than the other three strains. Third, we analyzed genes related to biocontrol activities, namely those encoding production of antifungal metabolites such as PCA and Plt. The genomic information indicated that the secondary metabolites differ markedly among the four PGPR. For example, GP72 contained putative gene clusters for biosynthesis of the siderophore Acr, whereas the other strains contained gene clusters for biosynthesis of different siderophores. Some rhizobacteria cannot produce antifungal compounds, but promote plant growth in the absence of pathogens. One such strain was *P*. *stutzeri* A1501, which fixes nitrogen. Therefore, the metabolic pathways, transporters, and regulators related to cell metabolism provide directions to improve plant growth-promoting activities. Genetic modification may accelerate the commercialization of PGPR as biocontrol agents, which could further contribute to sustainable development of agriculture.

## Methods

### Medium and growth conditions for *P*. *chlororaphis* GP72

*P*. *chlororaphis* GP72 (deposited in China General Microbiological Culture Collection Center; collection number 1748), isolated from green pepper rhizosphere in eastern China, was incubated at 28°C in King’s medium B [177].

### Genome sequencing and annotation

The genome of *P*. *chlororaphis* GP72 was sequenced using the Illumina GAIIx platform and assembled using VELVET 1.1.07. The genome of GP72 was automatically annotated using the RAST server [178], and proceeded with manual curation and comparative analysis using the IMG/ER system (https://img.jgi.doe.gov/cgi-bin/er/main.cgi) [179]. The genome sequence is available at the IMG database [39]. Information of COGs [40], combined with that from the Conserved Domain Database, was also used in the comparisons. The metabolic pathways were examined using KAAS (KEGG Automatic Annotation Server) [180] and the MetaCyc database [181].

### Nucleotide sequence accession number

This whole genome shotgun project has been deposited in DDBJ/EMBL/GenBank under the accession number AHAY00000000.

### Genome comparisons

The genome sequence of GP72 was aligned against sequences of other *Pseudomonas* genomes from NCBI’s Entrez database and the IMG database. Pair-wise alignments were performed using WebACT (http://www.webact.org/WebACT/home) [41]. BLAST atlases [42] were generated using the CBS DTU online tool, GeneWiz browser 0.94 server (http://www.cbs.dtu.dk/services/gwBrowser/). Strain-specific and conserved genes were identified using the mGenomeSubtractor web server (http://bioinfo-mml.sjtu.edu.cn/mGS/) [43]. The conserved CDSs were identified using a homology (H) value cut-off of 0.42 at E-value <10^-5^. Comparative genomic analyses of GP72, Pf-5, M18, and A1501 were conducted using the tool set available at the IMG website; genes homologous to those in GP72 were computed with an E-value < 10^-2^ and at 60% identity; BLAST comparisons between PGPR and *P*. *stutzeri* A1501 were screened at the 30% identity threshold.

### Phylogenetic analysis

The phylogenetic relationships among completely sequenced *Pseudomonas* were determined by a multilocus sequence analysis using a concatenated data set of *gyrB* and *rpoD* genes. Multiple-sequence alignments were carried out with Clustal W (http://www.genome.jp/tools/clustalw/) [182]. Evolutionary distances were calculated using the neighbor-joining method [183] with 1000 bootstrap replicates, using Phylip 3.67 software (http://evolution.genetics.washington.edu/phylip.html). The phylogenetic tree was generated using interactive tree of life (iTOL) software [184].

## Abbreviations

PGPR: Plant growth-promoting rhizobacteria; COG: Clusters of Orthologous Groups; Plt: Pyoluteorin; Prn: Pyrrolnitrin; PCA: Phenazine-1-carboxylic acid; 2-OH-PHZ: 2-Hydroxyphenazine; HCN: Hydrogen cyanide; IAA: Indole-3-acetic acid; PYO: Pyocyanin; Pvd: Pyoverdin; Pch: Pyochelin; ACT: Artemis Comparison Tool; DO: Dioxygenase; Tyr: Tyrosine; HPP: 4-Hydroxyphenylpyruvate; HPC: Homoprotocatechuate (3,4-Dihydroxyphenylacetate); HPCD: 3,4-Dihydroxyphenylacetate 2,3-dioxygenases; ROS: Reactive oxygen species; PHA: Polyhydroxyalkanoate; PQQ: Pyrrolquinoline quinine; GB: Glycine betaine; IVET: in vivo expression technology; ABC transporter: ATP binding cassette transporter; MFS: Major facilitator superfamily; TRAP-T: Tripartite ATP-independent periplasmic transporter; PTS: Phosphotransferase system; GntT: Guconate transporter; LysE family: Lysine exporter family; RhtB family: Resistance to homoserine/threonine family; APC family: Amino acid-polyamine-organocation family; DMT family: Drug/metabolite transporter family; RND family: Resistance-nodulation-cell-division family; MATE family: Multidrug and toxic compound extrusion family; SMR family: Small multidrug resistance family; Fit: *P*. *fluorescens* insecticidal toxin; Mcf: Makes caterpillars floppy; Acr: Achromobactin; ACC: 1-Aminocyclopropane-1-carboxylate; iTOL: Interactive tree of life

## Competing interests

The authors declare that they have no competing interests.

## Authors’ contributions

XHZ, XMS, HBH, and HSP conceived, coordinated, and designed the study. WW, XHZ and XMS were responsible for sequencing, finishing, and annotating. XMS, XHZ, and HSP performed experiments and data analyses. XHZ, XMS, and HBH drafted the manuscript. All authors read and approved the final manuscript.

## Supplementary Material

Additional file 1 Comparison of putative genes related to peripheral pathways for aromatic catabolism among pseudomonad PGPR. Click here for file

Additional file 2 Putative genes involved in tolerance to antibiotics, osmotic-, and temperature-shock in the pseudomonad PGPR genomes. Click here for file

Additional file 3 Comparison of putative genes related to attachment to plant rhizosphere among pseudomonad PGPR. Click here for file

Additional file 4 Comparison of putative genes involved in nitrogen fixation and denitrification process among pseudomonad PGPR. Click here for file
